# A Comparative Study of Same-Session Laparoscopic Cholecystectomy (LC) and Endoscopic Retrograde Cholangiopancreatography (ERCP) in Cholecystocholedocholithiasis: LC-First Versus ERCP-First Approach

**DOI:** 10.7759/cureus.106437

**Published:** 2026-04-04

**Authors:** Ahmed A Ateik, Saif A Ghabisha, Faris Alhajami, Tofik A Almekhlafi

**Affiliations:** 1 Department of Surgery, 21 September University for Medical and Applied Sciences, Sana’a, YEM; 2 Department of General Surgery, Ibb University, Ibb, YEM; 3 Department of Surgery, Faculty of Medicine, Sana’a University, Sana’a, YEM; 4 Department of Surgery, Faculty of Medicine and Health Sciences, Sana’a University, Sana’a, YEM

**Keywords:** cholecystectomy, cholecystocholedocholithiasis, choledocholithiasis, common bile duct stones, endoscopic retrograde cholangiopancreatography (ercp), laparoscopic cholecystectomy, same-session procedure

## Abstract

Background

Cholecystocholedocholithiasis (CCL) presents significant management challenges. The optimal sequencing of laparoscopic cholecystectomy (LC) and endoscopic retrograde cholangiopancreatography (ERCP) when performed during the same operative session remains controversial. This study compared the efficacy, safety, and clinical outcomes of two same-session approaches: LC-first versus ERCP-first.

Patients and methods

A retrospective comparative study was conducted at Yemen Germany Hospital, Sana’a, Yemen, between January 2017 and August 2022. A total of 150 patients were included. Group A (n = 80) underwent ERCP-first followed by LC, while Group B (n = 70) underwent LC-first followed by intraoperative ERCP. All procedures were performed by a surgeon-led team with expertise in advanced laparoscopy and therapeutic ERCP. Primary outcomes included successful common bile duct (CBD) stone clearance, operative time, and conversion to open surgery. Secondary outcomes included technical difficulty, complications, hospital stay, and cost. Statistical significance was set at p < 0.05.

Results

Successful CBD stone clearance was achieved in 70 patients (87.5%; 95% CI: 78.5%-93.3%) in Group A and 67 patients (95.7%; 95% CI: 88.1%-98.9%) in Group B (p = 0.098). Mean total operative time was significantly shorter in Group B (69.8 ± 22.1 minutes) compared with Group A (83.3 ± 13.5 minutes; p < 0.001). The mean LC duration was also shorter in Group B (38.4 ± 13.2 vs. 41.2 ± 8.98 minutes; p = 0.046), while ERCP duration did not differ significantly (37.4 ± 11.4 vs. 43.3 ± 11.8 minutes; p = 0.122). Median hospital stay and estimated cost were also lower in Group B (1 vs. 3 days and $1,100 vs. $1,300; p < 0.001 and p = 0.04, respectively). Conversion to open surgery occurred in seven patients (8.8%; 95% CI: 3.6%-17.2%) in Group A and two patients (2.9%; 95% CI: 0.3%-9.9%) in Group B (p = 0.17). Technical difficulty due to bowel distension was observed only in Group A (four patients; 5.0%; 95% CI: 1.4%-12.3%; p = 0.12). Postoperative pancreatitis occurred in four patients (5.0%; 95% CI: 1.4%-12.3%) in Group A and two patients (2.9%; 95% CI: 0.3%-9.9%) in Group B (p = 0.68).

Conclusions

Both sequencing strategies were safe and effective when performed by an experienced, surgeon-led team. The LC-first approach was associated with shorter operative time, specifically due to a shorter LC duration, reduced hospital stay, and lower cost, while maintaining comparable stone clearance rates. These findings suggest that, in settings with coordinated surgical-endoscopic expertise, the LC-first approach may be preferable. Prospective randomized studies are needed to confirm these findings and evaluate patient-reported outcomes.

## Introduction

Choledocholithiasis complicates 10%-20% of patients with symptomatic gallstone disease. While some common bile duct (CBD) stones remain asymptomatic, others may lead to life-threatening complications, including acute cholangitis, severe pancreatitis, and obstructive jaundice [1]. Approximately 90% of CBD stones originate in the gallbladder; however, stones impacted at the cystic duct or gallbladder neck may not migrate and can pose distinct surgical challenges. Nevertheless, cholecystocholedocholithiasis (CCL) requires definitive management of both the gallbladder and the bile duct stones [2,3].

For decades, the standard management of CCL has been a two-stage approach, typically involving preoperative endoscopic retrograde cholangiopancreatography (ERCP) with stenting for ductal clearance, followed by interval laparoscopic cholecystectomy (LC) [3,4]. Although this strategy is widely adopted and endorsed by international guidelines, it has several limitations, including prolonged total hospital stay, multiple anesthetic exposures, higher healthcare costs, and reduced patient convenience [5-9]. These limitations may be addressed by performing endoscopic CBD stone clearance during the same operative session as LC [4]. However, this approach requires close coordination between the surgeon and the endoscopist [10]. Ibrarullah et al., drawing on extensive experience in laparoscopy and endoscopy, adopted this model and concluded that, in selected patients, LC followed by endoscopic CBD clearance is safe and effective [7].

Within the framework of single-stage management, a key clinical question remains: what is the optimal procedural sequence when ERCP and LC are performed during the same operative session? Current strategies include the ERCP-first approach (ERCP followed immediately by LC) and the LC-first approach (LC followed immediately by ERCP) [11]. The ERCP-first approach allows for biliary decompression and ductal clearance prior to surgery [9]. In contrast, the LC-first approach enables early cystic duct control, which may reduce the risk of intraoperative stone migration, and avoids bowel distension caused by endoscopic air insufflation, which can impair laparoscopic visualization of Calot’s triangle [12].

At present, the choice of procedural sequence is often guided by a tailor-made approach based on patient-specific factors and local expertise, rather than high-level comparative evidence [13]. Direct comparative evidence within the single-stage framework remains limited, as most studies have focused on comparisons between single-stage and two-stage approaches. Preliminary data suggest that the LC-first approach may be associated with shorter operative times [8-10,12]; however, robust comparative studies are lacking. This evidence gap is particularly relevant in resource-limited settings, where optimizing efficiency and minimizing the need for re-intervention are critical.

Therefore, this study aimed to compare the clinical outcomes, procedural efficiency, and safety of ERCP-first versus LC-first sequences in the single-stage management of CCL. Specifically, we evaluated total operative time, CBD clearance success, postoperative complications, conversion to open surgery, and length of hospital stay. We hypothesized that the LC-first approach would be associated with shorter operative times and reduced hospital stay, without compromising safety or efficacy.

## Materials and methods

Study design and setting

This retrospective comparative study was conducted at Yemen Germany Hospital, Sana’a, Yemen, between January 2017 and August 2022. All procedures (both ERCP and LC) were performed by the same surgeon (A.A.), who has 10 years of experience and is fully trained in advanced laparoscopy and therapeutic ERCP. This ensured a standardized, surgeon‑led combined approach.

The study protocol was approved by the Medical Research Ethics Committee of 21 September University for Medical & Applied Sciences (approval number: S-98-H-02-F24) and complied with the Declaration of Helsinki. Given the retrospective study design, the requirement for written informed consent was waived. Patient confidentiality was maintained through data anonymization.

Sample size calculation

The sample size was calculated based on the mean difference in total operative time between the ERCP‑first and LC‑first approaches reported in a contemporary comparative study by Sayed et al. [14], which found a difference of 6.8 minutes (standard deviation (SD) approximately 17.8 minutes). Assuming a two-sided significance level of 5% and 80% power, the required sample size was 108 patients per group. After accounting for a potential 10% attrition rate, the target was set at 119 patients per group (total 238 patients). However, due to the retrospective nature of the study and the available patient pool during the study period, a total of 150 consecutive patients (80 in Group A and 70 in Group B) were included. Consequently, the study is underpowered to detect a difference as small as 6.8 minutes, and the analysis should be considered exploratory; this, in turn, resulted in reduced statistical power to detect the hypothesized effect size.

Participants

The study included 150 eligible patients with symptomatic CCL diagnosed by preoperative imaging. The inclusion criteria were: adult patients aged ≥18 years; concomitant gallbladder and CBD stones confirmed by ultrasonography (US), magnetic resonance cholangiopancreatography (MRCP), or intraoperative cholangiography (IOC); CBD stone size 5-15 mm (largest diameter); and American Society of Anesthesiologists (ASA) physical status I-III. Exclusion criteria included acute cholangitis or severe acute pancreatitis requiring urgent intervention; altered upper gastrointestinal anatomy (e.g., gastric bypass, Billroth II, Roux‑en‑Y); suspected gallbladder malignancy; and pregnancy. Patients with CBD stones larger than 15 mm were excluded from the study and underwent laparoscopic CBD exploration instead.

Distribution of treatment groups and sequence of intervention

Allocation was non‑randomized and based on institutional practice trends. From 2017 to 2019, the Division followed an ERCP‑first protocol (Group A), transitioning to an LC‑first protocol in 2020, due to advances in surgical practice (Group B). This temporal shift introduces potential confounding in the form of a learning curve and improvements in perioperative management, which are addressed in the Discussion section.

Patients in Group A underwent ERCP in the semi‑prone position, followed by same‑session LC in the supine position. Patients in Group B underwent LC prior to immediate intraoperative ERCP. In both groups, failed ERCP resulted in conversion to laparoscopic or open CBD exploration.

Methods and procedures

**Figure 1:**
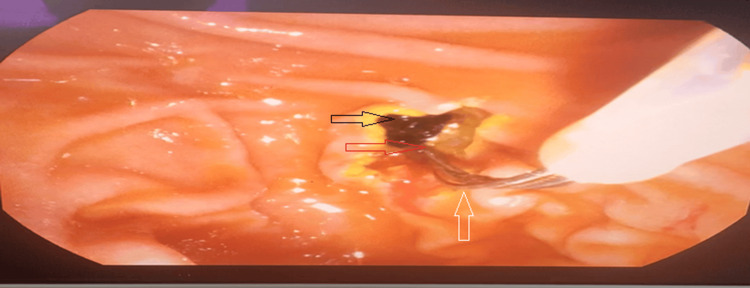
ERCP Stone Extraction Endoscopic view of the major duodenal papilla during endoscopic retrograde cholangiopancreatography (ERCP). Following sphincterotomy (red arrow), a pigmented biliary stone (black arrow) is extracted from the common bile duct into the duodenal lumen, using a balloon catheter (white arrow).

These approaches and techniques are described as follows. Cannulation of the bile duct was achieved using a wire‑guided technique, with minimal pancreatic duct manipulation, followed by sphincterotomy and stone extraction using balloons or baskets (Figure 1). ERCP procedure time was measured from duodenoscope insertion to stone extraction. If ductal clearance was not achieved, a plastic biliary stent was placed.

**Figure 2:**
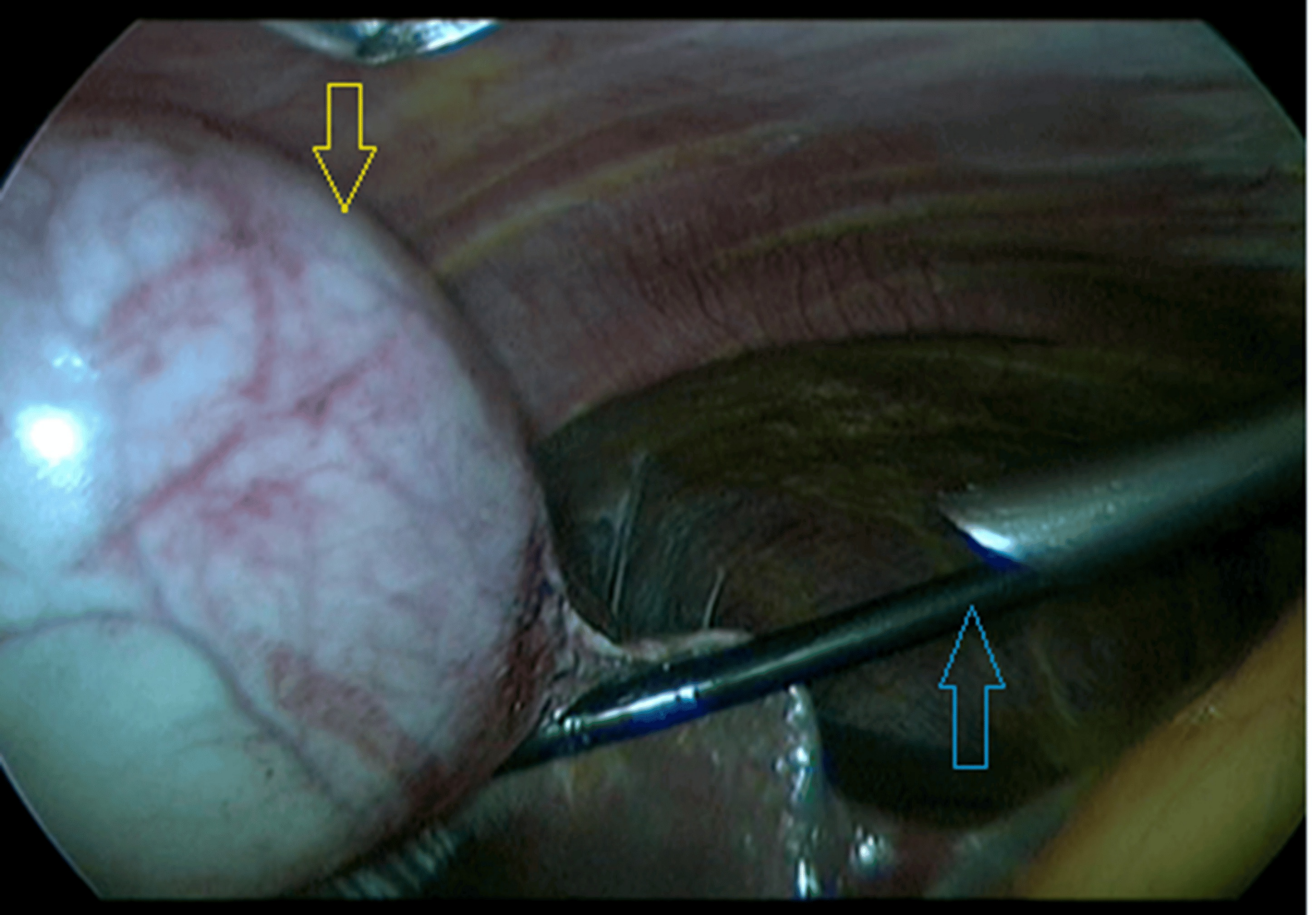
Laparoscopic View Following ERCP Intraoperative laparoscopic image demonstrating a distended gallbladder (yellow arrow) during cholecystectomy performed after endoscopic retrograde cholangiopancreatography (ERCP). A laparoscopic grasper (blue arrow) provides cephalad traction on the gallbladder fundus, to facilitate exposure of the cystic structures. This image illustrates the impact of bowel distension following ERCP on laparoscopic visualization.

LC was performed using a four‑port technique under CO₂ pneumoperitoneum (12-14 mmHg). The critical view of safety was established before clipping the cystic duct and artery, and the gallbladder was removed via the epigastric port. LC operative time was measured from skin incision to closure (Figure 2). Total operative time was calculated as the sum of LC duration and ERCP duration, including any patient repositioning time.

Outcome measures

The primary endpoints were successful stone clearance (confirmed by completion cholangiogram), total operative time (from skin incision to closure after both procedures), and conversion to open surgery. Secondary outcomes included technical difficulty during LC (e.g., poor visualization due to bowel distension), difficult ERCP cannulation requiring precut sphincterotomy, postoperative complications (pancreatitis, bleeding, perforation, or bile leak), residual stone rates, length of hospital stay (defined as the number of nights spent in the hospital after the procedure, requiring hospitalization or prolongation of planned admission), and estimated hospitalization costs.

Definitions

Complete clearance was defined as the absence of filling defects on completion of the cholangiogram. Post‑ERCP pancreatitis (PEP) was defined according to consensus criteria as new‑onset abdominal pain, with serum amylase or lipase levels >3‑fold the upper limit of normal at 24 hours post‑procedure, that required hospitalization or prolonged an existing admission. Residual stones were defined as stones detected on postoperative imaging within 30 days.

Estimated hospitalization costs included direct medical expenses (operating room, supplies, medications, and bed charges). Currency fluctuations and indirect costs were not considered; therefore, these calculations should be interpreted as indicative rather than absolute. As this was a retrospective study, outcome assessors were not blinded to group allocation.

Statistical analysis

Data were analyzed using IBM SPSS Statistics for Windows, Version 26 (Released 2018; IBM Corp., Armonk, NY, USA). Normality was assessed using the Shapiro-Wilk test. Normally distributed variables were reported as mean ± SD, while non‑normally distributed data were presented as median and interquartile range (IQR). Categorical variables were compared using the chi‑square test or Fisher’s exact test, and continuous variables were analyzed using independent t‑tests or Mann-Whitney U tests, as appropriate based on normality assessment.

Given the exploratory nature of the study, no adjustment for multiple comparisons was performed; therefore, p‑values should be interpreted with caution. Missing data (present in <5% of variables) were handled using pairwise deletion. A sensitivity analysis using complete case analysis was performed and yielded consistent results. All tests were two‑tailed, and p < 0.05 was considered statistically significant.

## Results

Baseline characteristics and procedural approach

Group A comprised 80 patients who underwent ERCP‑first, followed by LC during the same session. Group B included 70 patients who underwent LC‑first, followed immediately by intraoperative ERCP. The two groups were well matched with respect to baseline demographic and clinical characteristics. The mean age was 50.2 ± 11.8 years in Group A and 48.5 ± 12.4 years in Group B (p = 0.39), with no statistically significant differences in sex distribution, ASA physical status, presenting symptoms, CBD stone size, or CBD diameter (Table 1).

**Table 1:** Baseline Demographic and Clinical Characteristics ^a^Independent samples t‑test; ^b^Chi‑square test SD: standard deviation; ASA: American Society of Anesthesiologists; CBD: common bile duct; ERCP: endoscopic retrograde cholangiopancreatography; LC: laparoscopic cholecystectomy

Characteristic	Group A: ERCP‑first (n = 80)	Group B: LC‑first (n = 70)	p‑value
Age (years), mean ± SD	50.2 ± 11.8	48.5 ± 12.4	0.39ᵃ
Male sex, n (%)	35 (43.8)	30 (42.9)	0.86ᵇ
ASA physical status, n (%)			0.74ᵇ
Class I-II	61 (76.3)	52 (74.3)	
Class III	19 (23.8)	18 (25.7)	
Presenting symptom, n (%)			0.80ᵇ
Biliary colic	65 (81.3)	58 (82.9)	
Obstructive jaundice	15 (18.8)	12 (17.1)	
CBD stone size (mm), mean ± SD	8.5 ± 2.4	8.2 ± 2.1	0.42ᵃ
CBD diameter (mm), mean ± SD	11.1 ± 2.2	10.8 ± 2.0	0.38ᵃ

Procedural efficacy and operative details

Both procedural sequences showed high rates of CBD stone clearance (Table 2). In Group A (ERCP‑first), endoscopic stone extraction was successful in 70 of 80 patients (87.5%; 95% CI: 78.5%-93.3%), with three patients (3.8%; 95% CI: 0.8%-10.6%) requiring biliary stent insertion. In Group B (LC‑first), stone extraction was successful in 67 of 70 patients (95.7%; 95% CI: 88.1%-98.9%), with three patients (4.3%; 95% CI: 0.9%-12.0%) requiring stent insertion. There was no statistically significant difference between groups (p = 0.098).

**Table 2:** Procedural Efficacy and Operative Outcomes ^a^Independent samples t‑test; ^b^Chi‑square test; ^c^Mann-Whitney U test (used for total operative time due to non‑normal distribution in one group; means shown for clinical interpretability). *Three patients in Group A required stent insertion with successful biliary drainage. Three patients in Group B also required stent insertion. CBD: common bile duct; ERCP: endoscopic retrograde cholangiopancreatography; LC: laparoscopic cholecystectomy; CI: confidence interval; SD: standard deviation

Variable	Group A: ERCP‑first (n = 80)	Group B: LC‑first (n = 70)	p‑value
Successful CBD stone clearance, n (%) (95% CI)	70 (87.5) (78.5-93.3)	67 (95.7) (88.1-98.9)	0.098ᵇ
Stent insertion required, n (%) (95% CI)*	3 (3.8) (0.8-10.6)	3 (4.3) (0.9-12.0)	-
Total operative time (minutes), mean ± SD (range)	83.3 ± 13.5 (68-130)	69.8 ± 22.1 (45-98)	<0.001ᶜ
ERCP duration (minutes), mean ± SD (range)	43.3 ± 11.8 (31-70)	37.4 ± 11.4 (21-82)	0.122ᵃ
LC duration (minutes), mean ± SD (range)	41.2 ± 8.98 (32-99)	38.4 ± 13.2 (21-71)	0.046ᵃ
Bowel distension (technical difficulty), n (%) (95% CI)	4 (5.0) (1.4-12.3)	0 (0.0) (0.0-5.1)	0.12ᵇ

The procedural sequence significantly affected operative duration. The mean total operative time in Group B (LC‑first) was 69.8 ± 22.1 minutes (range 45-98), which was significantly shorter than 83.3 ± 13.5 minutes (range 68-130) in Group A (p < 0.001). The mean LC duration was also significantly shorter in Group B (38.4 ± 13.2 vs. 41.2 ± 8.98 minutes; p = 0.046), while ERCP duration did not differ significantly (37.4 ± 11.4 vs. 43.3 ± 11.8 minutes; p = 0.122).

Complications and re‑interventions

Conversion to open surgery occurred more frequently in Group A, affecting seven patients (8.8%; 95% CI: 3.6%-17.2%). This included three cases of failed ERCP requiring open cholecystectomy with CBD exploration, and four cases of severe bowel distension due to endoscopic air insufflation, which impaired laparoscopic visualization. In Group B, two patients (2.9%; 95% CI: 0.3%-9.9%) required conversion to open surgery due to failed cannulation requiring open CBD exploration (p = 0.17, Fisher’s exact test).

PEP occurred in four patients (5.0%; 95% CI: 1.4%-12.3%) in Group A, and in two patients (2.9%; 95% CI: 0.3%-9.9%) in Group B (p = 0.68). All cases were managed conservatively; one case in Group A was classified as severe and required prolonged hospitalization. No cases of clinically significant bleeding, duodenal perforation, or bile leak were observed in either group (Table 3).

**Table 3:** Postoperative Complications, Hospital Stay, and Cost Analysis ^b^Chi‑square test; ^c^Mann-Whitney U test. *Three patients in Group A had failed ERCP requiring open cholecystectomy and CBD exploration; the other four conversions in Group A were due to bowel distension. ERCP: endoscopic retrograde cholangiopancreatography; LC: laparoscopic cholecystectomy; USD: United States dollars; IQR: interquartile range; CI: confidence interval

Variable	Group A: ERCP‑first (n = 80)	Group B: LC‑first (n = 70)	p‑value
Difficult cannulation during ERCP, n (%)	3 (3.8)	2 (2.9)	-
Conversion to open surgery, n (%) (95% CI)*	7 (8.8) (3.6-17.2)	2 (2.9) (0.3-9.9)	0.17ᵇ
Post‑ERCP pancreatitis, n (%) (95% CI)	4 (5.0) (1.4-12.3)	2 (2.9) (0.3-9.9)	0.68ᵇ
Re‑ERCP required (residual stones), n (%) (95% CI)	3 (3.8) (0.8-10.6)	0 (0.0) (0.0-5.1)	0.25ᵇ
Length of hospital stay (days), median (IQR)	3 (3-4)	1 (1-2)	<0.001ᶜ
Estimated cost per patient (USD), median (IQR)	1,300 (1,200-1,400)	1,100 (1,000-1,200)	0.04ᶜ

A second postoperative ERCP (re‑ERCP) was required in three patients (3.8%; 95% CI: 0.8%-10.6%) in Group A, whereas no patients in Group B required re‑intervention (0.0%; 95% CI: 0.0%-5.1%; p = 0.25, Fisher’s exact test).

Recovery and economic outcomes

The LC‑first approach was associated with a significantly shorter hospital stay, with a median of one day (IQR: 1-2) compared to three days (IQR: 3-4) in the ERCP‑first group (p < 0.001). Additionally, the LC‑first sequence was associated with lower estimated hospitalization costs, with a median cost of $1,100 (IQR: $1,000-$1,200) compared to $1,300 (IQR: $1,200-$1,400) in the ERCP‑first group (p = 0.04) (Table 3).

## Discussion

In this study, we compared two procedural sequences for single‑session management of concomitant CCL: the ERCP‑first approach followed by LC, versus the LC‑first strategy followed by intraoperative ERCP. We found that both approaches achieved high rates of CBD clearance, with acceptable complication profiles. However, the LC‑first sequence was associated with shorter operative time, lower conversion rates to open surgery, shorter hospital stay, and reduced cost. These results add to a growing body of evidence favouring an LC‑first, single‑stage approach, especially when the operation is conducted by a surgeon with combined laparoscopic and endoscopic expertise [7,14-17].

Previous studies have consistently reported CBD clearance success rates of 93%-100% for single‑session combined ERCP and LC, with no significant difference based on procedural sequence [13,18-20]. Sayed et al. reported similar findings (95.7% vs. 88.2%) in a comparable population [14]. In our study, CBD clearance rates were high in both groups (95.7% vs. 87.5%), with a slightly higher rate in the LC‑first group, although the difference was not statistically significant.

However, the 12.5% failure rate observed in the ERCP‑first group in our study necessitated stent placement or conversion to open CBD exploration. This raises the possibility that performing ERCP before cholecystectomy may delay the recognition of unfavorable anatomy or large, impacted stones until after endoscopic maneuvering has been completed [19]. Conversely, the LC‑first strategy allows early access to the cystic duct, facilitating the use of the rendezvous cannulation technique. Previous reports corroborate this benefit, demonstrating higher cannulation success rates and lower PEP rates with this approach [13,16,21].

In our study, total operative time was significantly shorter in the LC‑first group (mean 69.8 ± 22.1 vs. 83.3 ± 13.5 minutes, p < 0.001). The mean LC duration was also shorter in the LC‑first group (38.4 ± 13.2 vs. 41.2 ± 8.98 minutes, p = 0.046), while ERCP duration did not differ significantly (37.4 ± 11.4 vs. 43.3 ± 11.8 minutes, p = 0.122). However, this finding should be interpreted with caution, given the limited sample size and the later timing of the LC‑first group, which may reflect increased surgeon experience; nevertheless, the magnitude of the difference is unlikely to be explained solely by a learning effect. Our findings are consistent with the randomized controlled trial by Sayed et al., who reported total operative times of 75.1 ± 19.3 vs. 81.9 ± 16.7 minutes for the LC‑first and ERCP‑first sequences, respectively [14]. Sahoo et al. similarly found that single‑stage rendezvous procedures were faster than two‑stage approaches [17]. This difference is likely multifactorial. First, performing ERCP before cholecystectomy causes bowel distension from air insufflation [7]. In our study, this was directly implicated in four of the seven conversions in the ERCP‑first group. Such distension can obscure the hepatocystic triangle and increase dissection difficulty. Second, completing the cholecystectomy first allows the surgeon to proceed to ERCP in a decompressed and unencumbered operative field [12]; although CO₂ insufflation during ERCP partially mitigates distension, it does not eliminate it.

Nevertheless, the LC‑first approach carries inherent trade‑offs. Committing to cholecystectomy before confirming successful CBD clearance risks losing cystic duct access for a rendezvous procedure if cannulation fails, potentially necessitating repeat ERCP or conversion to open surgery [17,22]. Additionally, this sequence often requires repositioning the patient from supine to prone or left lateral decubitus (or performing ERCP in the challenging supine position), which adds time and demands specialized expertise.

While the conversion rate was numerically higher in the ERCP‑first group (8.8% vs. 2.9%) in our study, this difference was not statistically significant. This trend parallels the results of Morino et al. (18% vs. 0%) [23], who found a significantly higher conversion rate after preoperative ERCP compared to rendezvous techniques. The physiologic effects of ERCP, which include air insufflation and fluid shifts, may predispose to difficulty performing the subsequent cholecystectomy laparoscopically.

A relevant consideration in evaluating the LC‑first approach is its facilitation of the laparoendoscopic rendezvous (LERV) technique. Although our study did not formally quantify the use of this technique, the LC‑first sequence inherently enables it. Several studies have demonstrated that the rendezvous technique reduces the incidence of PEP compared with preoperative ERCP: Morino et al. reported rates of 2% versus 15% [23], Lella et al. reported 0% versus 10% [15], and Koiava et al. reported a PEP rate of 3.75%, with 100% CBD clearance, in a series of 80 patients undergoing LERV [24]. The mechanism is well characterized: guidewire passage through the cystic duct into the duodenum provides a defined path for cannulation, reducing repeated attempts at the papilla and minimizing trauma to the pancreatic orifice [16,25]. Our observation of a lower PEP rate in the LC‑first group (2.9% vs. 5.0%), though not statistically significant, is consistent with this proposed mechanism.

Despite these favorable findings, the LC‑first approach is not universally adopted. The primary barrier is logistical: coordinating laparoscopic and endoscopic teams in a single operative session can be challenging in institutions where endoscopy suites and operating rooms are separate, leading to scheduling inefficiency [26]. Additionally, some evidence suggests that the two‑stage approach (ERCP followed by LC) remains safe and effective. A meta‑analysis by Lyu et al. found that, while single‑stage approaches reduced hospital stay and cost, overall morbidity and mortality did not differ significantly from those of two‑stage management [9]. Patient selection also remains important, as emphasized by early reports on same‑session procedures [7,27].

Our study addresses this logistical concern by employing a dual‑skilled surgeon model, which eliminates the need for inter‑team coordination. This integrated approach is gaining recognition and has been validated in larger series, including La Barba et al.'s report of 200 patients treated with a single‑team laparoendoscopic approach, with favorable outcomes [28].

In this study, there were no residual stones requiring reintervention in the LC‑first group (0% vs. 3.8% in the ERCP‑first group). Although not statistically significant, this may reflect a benefit of performing ERCP after cholecystectomy, which prevents stone migration from the gallbladder to the CBD in the interval between procedures [14].

The LC‑first group also experienced a shorter hospital stay (one vs. three days) and reduced cost ($1,100 vs. $1,300). These findings are in line with the economic evaluation by Garbarini et al., who observed a mean cost reduction of approximately €1,000 per patient with the LERV approach compared to two‑stage management, primarily as a result of shorter length of stay [29], and with Poulose et al., who reported that single‑stage management was the cost‑dominant option for choledocholithiasis [30].

Study limitations

This study has several limitations. First, the retrospective, non‑randomized design introduces selection and information bias. The ERCP‑first approach predominated in the earlier study period, and the LC‑first approach in the later period, confounding outcomes with the learning curve and evolving perioperative care over the five‑year interval; a sensitivity analysis by year was not feasible given the sample size. Second, the single‑center, single‑surgeon design limits generalizability, as outcomes may differ in settings where ERCP is performed by separate gastroenterology teams. Third, the absence of multivariate regression analysis precludes adjustment for potential confounders, such as age, stone size, and ASA classification. Fourth, the final sample size (80 in Group A and 70 in Group B) fell short of the target enrollment of 108 patients per group (238 total) calculated from the literature [14], likely underpowering the study for the detection of differences in operative time as small as 6.8 minutes and for rare secondary outcomes. Fifth, cost estimates are based on institutional charges and may not be generalizable across different reimbursement models. Finally, long‑term follow‑up and patient‑reported outcome measures were not collected, limiting our understanding of late complications, stone recurrence, and patient experience.

## Conclusions

In this study, the LC‑first approach to single‑session management of CCL was associated with shorter total operative time (specifically due to a shorter LC duration) and shorter hospital stay, compared with the ERCP‑first approach, without a significant difference in ductal clearance or complication rates. Given the retrospective design and limited sample size, these findings should be considered hypothesis‑generating. For centers with a dedicated, integrated, surgeon‑led endoscopy team, the LC‑first sequence may represent a feasible alternative; however, patient selection remains essential, and the approach should be tailored to individual clinical factors and local expertise. Prospective, randomized, multicenter trials are needed to establish the optimal procedural sequence and evaluate patient‑reported outcomes.
